# The Influence of Suprascapular Notch Shape on the Visualization of Structures in the Suprascapular Notch Region: Studies Based on a New Four-Stage Ultrasonographic Protocol

**DOI:** 10.1155/2017/5323628

**Published:** 2017-12-20

**Authors:** Hubert Jezierski, Michał Podgórski, Ludomir Stefańczyk, David Kachlik, Michał Polguj

**Affiliations:** ^1^Department of Orthopedics and Traumatology, Ministry of the Interior Hospital, Północna Str. 42, 91-245 Łódź, Poland; ^2^Department of Normal and Clinical Anatomy, Interfaculty Chair of Anatomy and Histology, Medical University of Łódź, Łódź, Poland; ^3^Department of Radiology and Diagnostic Imaging, Medical University of Łódź, Kopcińskiego 22, 90-153 Łódź, Poland; ^4^Department of Anatomy, Second Faculty of Medicine, Charles University, U Nemocnice 3, 128 00 Praha 2, Czech Republic; ^5^Department of Angiology, Interfaculty Chair of Anatomy and Histology, Medical University of Łódź, Łódź, Poland

## Abstract

Evaluation of the morphology of the suprascapular notch region is important from a clinical point of view because it is the most common site of suprascapular nerve compression and injury. A group of 120 patients underwent ultrasound examination of the suprascapular notch region according to our original four-stage “step-by-step” protocol. The notches were classified based on their morphology and measurements like maximal depth (MD) and superior transverse diameter (STD) as follows: type I-MD is longer than STD, type II-MD and STD are equal, type III-STD is longer than MD, and in type IV/V-notches only the bony margin was visualized without depression. Both suprascapular notches were fully visualized in 115 of 120 patients. The type III suprascapular notch was the most prevalent (64.2%), followed by type IV/V (18.7%), type I (11.1%), and type II (6.0%). Color Doppler analysis allowed the suprascapular artery to be recognized in all visualized notches. The suprascapular vein was visible in 176 notches and the suprascapular nerve in 150. Notches containing both suprascapular nerve and vein were significantly wider and shallower than average. As the suprascapular artery is the most easily recognised structure in the area, it may serve as a useful landmark of the suprascapular notch.

## 1. Introduction

The aim of Clinical Anatomy as a discipline is to apply pure anatomical knowledge for clinical goals concerning diagnostics and treatment, and its growth has been hastened by the development of modern diagnostic techniques. For the visualization of small, superficially situated structures, it is both helpful and practical to apply high-frequency ultrasound (US) transducer technology [1, 2]. The availability, cheapness, and repeatability of ultrasound examination are known to offer significant advantages as a means of visualization, and the use of ultrasound in regional anaesthesia interventions has been found to result in a greater success rate with a shorter application time and the possibility of avoiding particular complications [3, 4].

**Figure 1:**
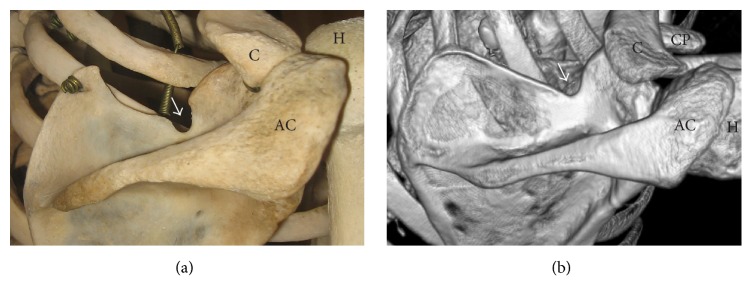
Posterior surface of the scapula: arrow: suprascapular notch, AC: acromion, C: clavicle, CP: coracoid process, and H: humerus. (a) The dry scapula; (b) three-dimensional volume rendering (VR) multidetector computed tomography (MDCT).

The suprascapular notch is a depression in the superior border of the scapula, medially to the coracoid process (Figure 1). The lateral and medial borders of the suprascapular notch are connected with the superior transverse scapular ligament, which usually passes below the suprascapular artery and above both the suprascapular vein and the suprascapular nerve [5].

The suprascapular notch and structures in its region are characterized by varied morphology [6, 7]. This variation is important from the clinical point of view. First, because it is the most common site of suprascapular nerve compression and injury [8, 9], and, second, because the morphological variations in this region may be a risk factor of these pathologies [10, 11].

The area of the suprascapular notch determines the space available for the nerve, and so plays a crucial role for predicting compression syndrome or trauma of the suprascapular nerve. Neuropathy of the suprascapular nerve is responsible for about 0.4–2% of shoulder girdle pain [8, 12]. This condition must be taken into consideration when differentiating the condition from others involving similar symptoms. It has considerable social significance despite being a rare pathology; it is typically found in professionally active men under 40. In most cases, diagnosis is made when supraspinatus and infraspinatus muscles are in the state of atrophy, which may cause the patient to have limited capacity to work or even cause permanent invalidity [8, 9, 12].

The aim of this study was to perform a four-stage ultrasound investigation of the suprascapular notch area. It is the first such detailed study to give a “step-by-step” description of this procedure. The second aim was to visualize the types of suprascapular notch and suprascapular nerve-vessel bundle based on our four-stage ultrasound protocol.

## 2. Materials and Methods

A total of 120 patients (66 women and 44 men) were recruited from the Orthopedics Department of the Ministry of Internal Affairs Hospital in Łódź. The research project was approved by the Bioethics Commission of the Medical University of Lodz (Protocol number ID: RNN/586/14/KE). In the study, all of the procedures that took place were in accordance with the ethical standards of the committee on human experimentation (institutional and national) and with the Helsinki Declaration of 1975, as revised in 2008. All participants gave informed oral and written consent. The exclusion criteria were as follows: injury, operation or deformations of the shoulder region; scapular fracture; active neoplasmatic disease with metastases to scapula; wounds and scars on the skin of the shoulders area.

A Toshiba memioXG (Toshiba, Japan) with a 5–10 MHz linear transducer was used for the sonographic examination of the suprascapular notch according to our newly developed protocol. The patient was placed in a sitting position in front of the researcher. Ultrasound investigation of the suprascapular notch region consisted of four steps.

**Figure 2:**
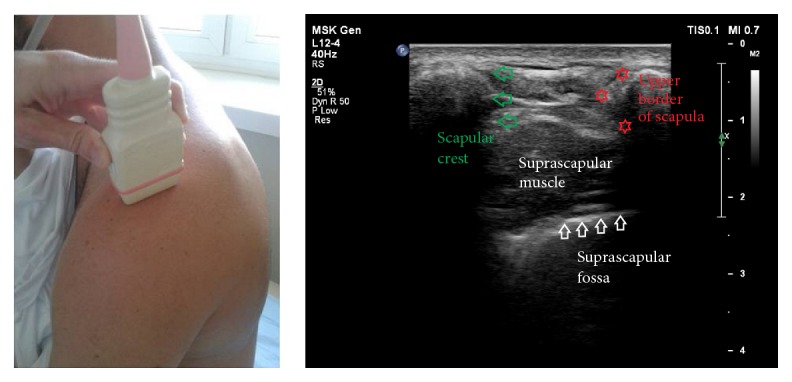
Ultrasonographic investigation of the suprascapular notch region (stage I of protocol).

*Step I*. The spine of the scapula was recognized and the transducer was placed superior to it in a parasagittal plane (Figure 2). In this position, a transverse section of the supraspinous fossa was visualized. Next, the supraspinous fossa region was investigated from medial to lateral. The aim of this step was to determine the boundaries formed by the spine of the scapula and the superior border of the scapula (Figure 2). 

**Figure 3:**
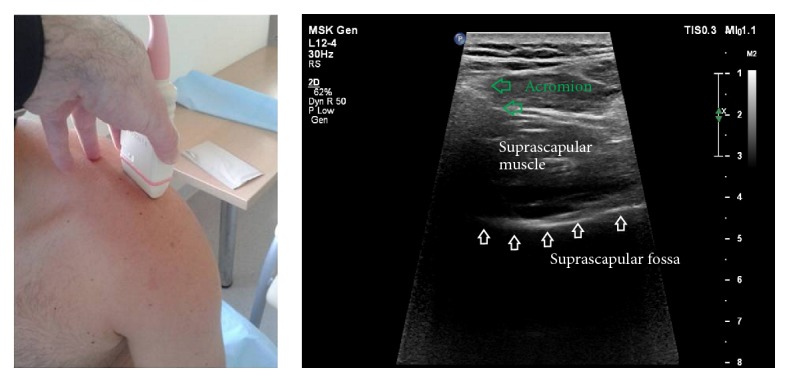
Ultrasonographic investigation of the suprascapular notch region (stage II of protocol).

*Step II*. The transducer was placed in a paracoronal plane, along the long axis of the supraspinatus muscle and parallel to the superior border of the scapula (Figure 3). Next, the supraspinous fossa region was investigated from medial to lateral to the point where the acromion obscured the supraspinatus muscle. The aim of this step was to determine whether the lateral border of the supraspinatus fossa floor can be visualized, and, if so, to identify this point. 

**Figure 4:**
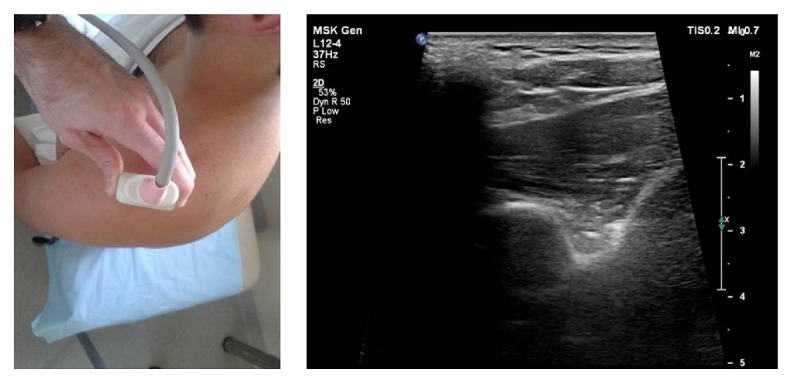
Ultrasonographic investigation of the suprascapular notch region (stage III of protocol).

*Step III*. The transducer was positioned at the end of the supraspinous fossa in the paracoronal plane. Next, the transducer was moved slowly in an anterior direction for visualization of the bony wall (anterior limitation) of the supraspinous fossa. In this position, the suprascapular notch was found (Figure 4). 

**Figure 5:**
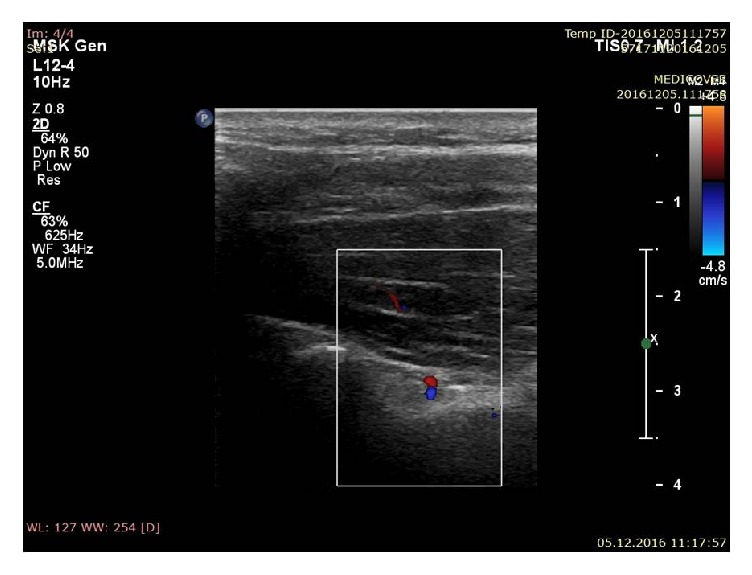
Ultrasonographic investigation of the suprascapular notch region (color Doppler) (stage IV of protocol); blue: suprascapular vein; red: suprascapular artery.

*Step IV*. After localization of the suprascapular notch, the color Doppler option was used to visualize the suprascapular vein and artery (Figure 5). The distinction between vein and artery was made on the basis of flow spectrum analysis.

### 2.1. Measurements of the Suprascapular Notch

During ultrasonographic investigation, the following measurements were collected:

- The superior transverse diameter (STD): the maximal distance in the horizontal plane between the corners of the suprascapular notch.
- The maximal depth (MD): the distance between the STD and the deepest point of the suprascapular notch measured in a plane perpendicular to the STD.

**Figure 6:**
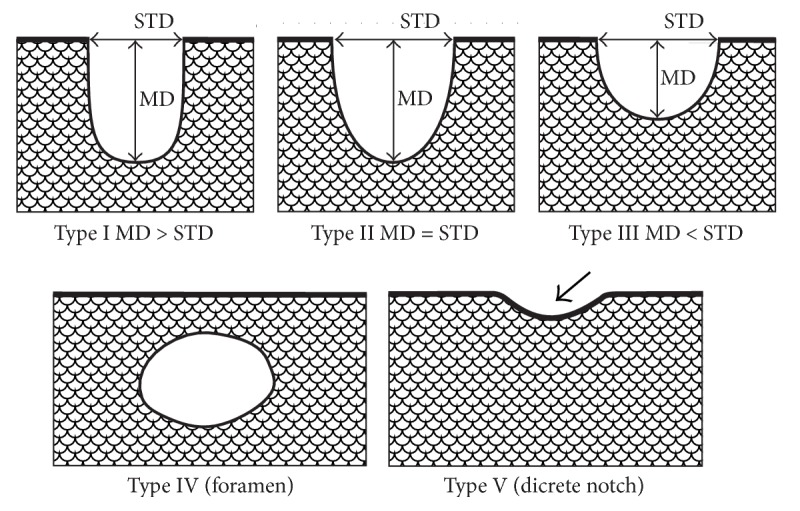
Schematic arrangement of the classification of the suprascapular notch variations. Type I: MD is longer than STD; Type II: MD and STD are equal; Type III: STD is longer than MD; Type IV: a suprascapular foramen with a bony foramen is present; Type V: a discrete notch is present (arrow). MD: maximal depth; STD: superior transverse diameter.

The shape of the suprascapular notch was classified according to Polguj et al. [13] (Figure 6).

### 2.2. Statistical Analysis

Statistical analysis of the ultrasound test results was performed with Statistica 12.0 software. Mean and standard deviation were provided for continuous variables, while contingency tables and Chi^2^ test were applied for comparing nominal variables between groups. The Shapiro-Wilk test was used to evaluate the normality of the distribution of data. The Mann–Whitney test was then employed to compare continuous, nonnormally distributed, variables between two groups. A *p* value of 0.05 or below was considered significant.

## 3. Results

Both suprascapular notches were fully visualized in 115 of the 120 patients and in 5 of the 120 patients, unilaterally. In the other five, the suprascapular notch was obscured by the clavicle and could not be examined. All suprascapular notches were classified into one of four types. As it was impossible to sonographically differentiate between the types IV and V, notches in which only the bony margin was visualized without depression were recorded as “type IV/V.”

In the entire group of subjects, the most prevalent category of suprascapular notch was type III (64.2% of cases), followed by type IV/V (18.7%) and type I (11.1% of cases). The least frequent was type II (6.0%). No significant differences were found between body sites regarding the notch type (*p* = 0.9512) (Table 1).

**Figure 7:**
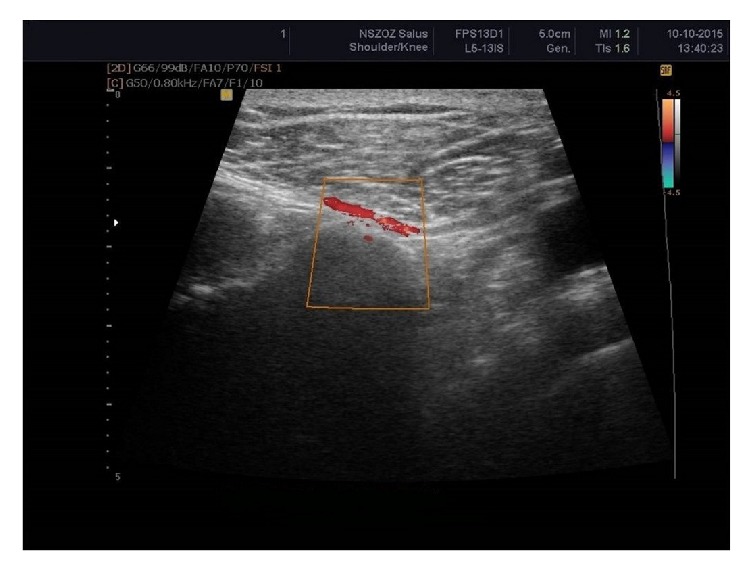
Ultrasonographic investigation of the suprascapular notch region (color Doppler) showing suprascapular artery (red).

**Figure 8:**
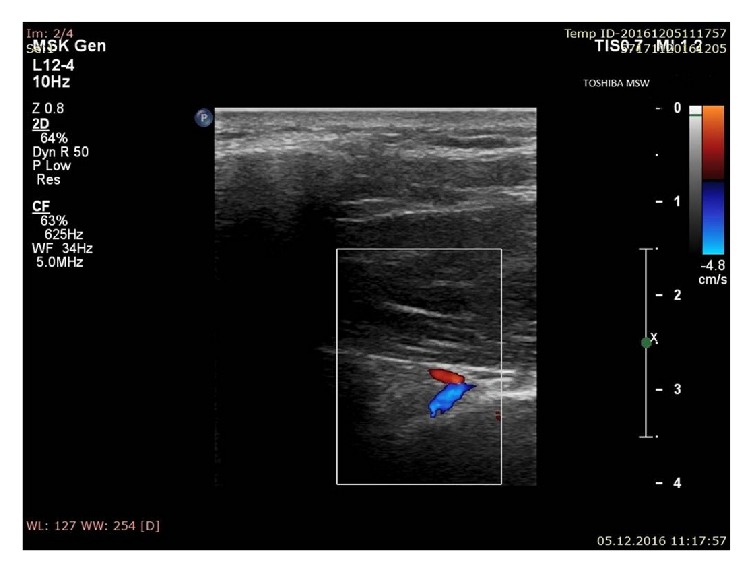
Ultrasonographic investigation of the suprascapular notch region (color Doppler); blue: suprascapular vein; red: suprascapular artery.

The suprascapular artery was recognized in all visualized notches with aid of the color Doppler scanning (*n* = 235; 100%) (Figure 7). The suprascapular vein was visible in 176 scapular notches (74.9%) (Figure 8). Among the suprascapular notches in which the vein was visible, type III (86.1%) and type IV/V (90.9%) predominated, which were more frequent in this group than the other types (*p* = 0.0001) (Table 2).

**Figure 9:**
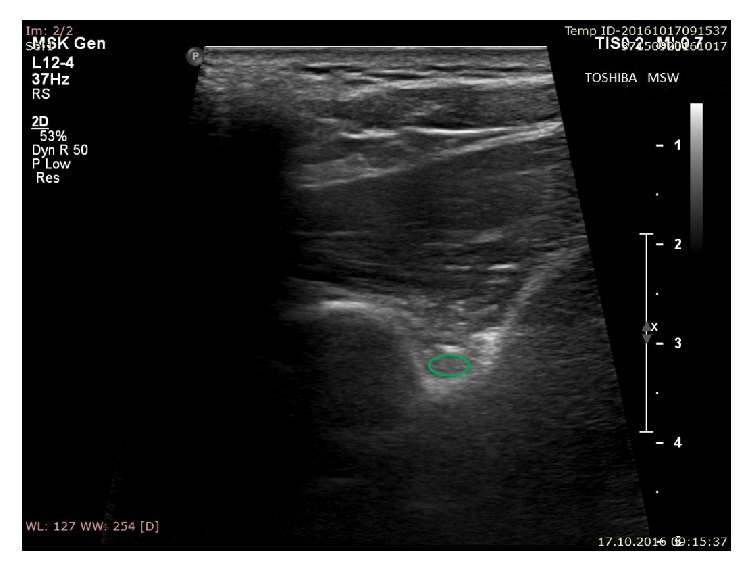
Ultrasonographic investigation of the suprascapular notch region. Green: suprascapular nerve.

The suprascapular nerve was identified in 150 notches (63.8%) (Figure 9). Among the suprascapular notches in which the nerve was located and measured, type IV/V predominated (86.36%) (*p* = 0.0001) (Table 3). Moreover, the notches other than the type IV/V in which the suprascapular nerve was found were wider (13.2 +/− 4.1 mm) and shallower (5.8 +/− 1.7 mm) than others (Table 4).

## 4. Discussion

In 1997, a study based on a group of 97 volunteers performed by Moriggl [14] first highlighted the value of ultrasound imaging in identifying the shape of the suprascapular notch, as well as in the general visualization of the suprascapular region. However, it was noted that interpretation was sometimes difficult when the superior transverse scapular ligament was partially ossified and impossible when complete ossification had taken place. Also Marhofer et al. [3] confirmed that visualization of the suprascapular nerve was limited when it was in close proximity to bony structures.

According to Harmon and Hearty [15], the final US probe position should be more in the coronal plane, because, in the transverse plane, the structures in the supraspinous fossa are all obscured by the spine of the scapula. In the procedure described by Okur et al. [16], the transducer was first positioned parallel to the spine of the scapula and then moved very slowly proximally until the supraspinous fossa was reached. In such a position, the transducer should be moved laterally to find the shape of the suprascapular notch. The suprascapular nerve was visualized as an ovoid hyperechoic structure approximately 40–50 mm deep under the skin.

Our new four-stage-protocol describes the “step-by-step” procedure of the visualization of the shape of the suprascapular notch, and of the suprascapular nerve and vessels. It combines two main positions, the parasagittal and paracoronal planes, along the supraspinous fossa. As an independent step, the color Doppler option was used to visualize the suprascapular vein and artery. This is a very important step because it allowed the suprascapular artery to be visualized in all patients with a visible suprascapular notch. We propose this vessel as a useful landmark for the recognition of the suprascapular notch during ultrasonographic investigation.

According to an ultrasonographic procedure proposed in 2010 by Peng et al. [17], the suprascapular nerve is better visualized in the coronal plane over the supraspinous fossa with a slight anterior tilt, on the floor of the spine of the scapula, between the spinoglenoid notch and the suprascapular notch. However, the authors note that the concave shape of the floor may be misinterpreted as the suprascapular notch: a finding confirmed in the present study. Therefore, to avoid mistakes in clinical practice, it is necessary to devise a method for the visualization of the suprascapular notch in accordance with a strict clearly described “step-by-step” procedure. Peng et al. [17] also note that the fascia of the supraspinatus muscle may sometimes be mistaken for the superior transverse scapular ligament.

From a practical point of view, the coracoid process, the spine of the scapula, and the acromion are thought to be very useful as landmarks in recognizing the position of the transducer during visualization of the suprascapular notch region [4, 17]. Rothe et al. [18] suggest that localization of the omohyoid muscle in a longitudinal section facilitates visualization of the SSN.

Ultrasonographic investigation may recognize not only the presence but also the shape of the suprascapular notches [4, 13]. According to Polguj et al. [13] this method is characterized by high specificity for the recognition of deep-shape suprascapular notches (97.8%) and high sensitivity in recognizing wide-shape notches (96.9%).

According to Yücesoy et al. [4], ultrasound allowed the artery-vein suprascapular complex at the suprascapular notch region to be visualized in 86 shoulders of 43 subjects (86%). In the present study, the distinction between vein and artery was made on the basis of flow spectrum analysis. Based on stage IV of our protocol, the suprascapular vein was visible in 176 of 235 cases (74.9%) and the suprascapular artery in all 235 cases (100%). However, visibility depended on the shape of the suprascapular notch. The suprascapular vein was more visible in wide and shallow notches (type III; 86.1%) than in narrow and deep ones (type I; 7.7%).

Gruber et al. [19] report that the suprascapular nerve could be visualized in 93.3% of healthy volunteers at the level of the omohyoid muscle and in 86.7% at the suprascapular notch. In our study, the suprascapular nerve was found in 63.8% of the 235 tested notches (*n* = 150); however, this frequency increased to 86.36% of cases in the type IV/V notches. Our findings also indicate that notches of types other than IV/V in which the suprascapular nerve was found tended to be wider and shallower than others. It is possible that shallow notches were more commonly presented in Gruber et al. [19].

Appropriate ultrasonographic visualization of the suprascapular notch and the suprascapular neurovascular bundle is necessary during suprascapular nerve block (SsNB). The first reports of SsNB were made by Wertheim and Rovenstine [20] in 1941 and by Milowsky and Rovenstine [21] in 1949. This procedure has since been widely used by anaesthetists in situations ranging from adhesive capsulitis to pain after shoulder arthroscopy. Harmon and Hearty [15] described it as a technique which can be easily learned and can be applied by emergency physicians with the support of ultrasound. Also Chang et al. [22] found that injection procedures were consistently more effective when applied with ultrasound guidance. Jeon et al. [23] note that the procedure requires the patient to be placed in a sitting position with the ultrasound probe placed horizontal to the scapular spine and the supraspinous fossa: the suprascapular notch is found by slowly locating the probe laterally, and the pulsating suprascapular artery is a good indicator for the location of the suprascapular nerve.

Ultrasound-guided suprascapular nerve block is a safe, effective, and accurate method for achieving immediate and long-term pain relief in patients with chronic, nonspecific shoulder pain, resulting in normal range of motion, normal imaging studies, and no identified shoulder pathology [24]. Previous studies support the use of suprascapular nerve block for treating hemiplegic shoulder pain in long-term chronic stroke patients [25]. In addition, a preliminary study by Okur et al. [16] suggests that ultrasound-guided blockage of the suprascapular nerve can hasten the recovery of range of motion in the shoulder following surgery in breast cancer survivors. Blockage of the suprascapular nerve might inhibit the transmission of pain arising from secondary scapulothoracic dysfunction, thereby promoting shoulder motion without interfering pain. It therefore represents a promising treatment approach for the rapid recovery of restricted shoulder motion in women with breast cancer before radiation treatment. Trabelsi et al. [26] found ultrasound-guided supraclavicular nerve block combined with ultrasound-guided suprascapular nerve block to be as effective as interscalene brachial plexus block for postoperative analgesia after shoulder instability surgery without decreasing potential side effects. A possible alternative to ultrasound-guided suprascapular nerve block may be arthroscopically guided nerve block. According to Ko et al. [27], when used in arthroscopic rotator cuff repair for medium-sized rotator cuff tears, arthroscopically guided suprascapular nerve block and blinded axillary nerve block provided a greater improvement in the visual analog scale for pain and greater patient satisfaction in the first 48 postoperative hours than blinded suprascapular nerve block.

Battaglia et al. [28] and Laumonerie et al. [29] report that the trajectory of the suprascapular nerve at the level of the suprascapular notch is deep, inconsistent, and in the vicinity of the suprascapular artery; these factors make ultrasound-guided procedures more challenging. Our four-stage procedure will allow better recognition of the suprascapular nerve and artery, thus preventing unexpected complications, such as bleeding, during ultrasound-guided suprascapular nerve block.

## 5. Conclusion

The shape and morphometry of the suprascapular notch affect the visualization of structures in the suprascapular notch region. The notches containing the suprascapular nerve and vein were significantly wider and shallower than average. The suprascapular artery, as the structure most often visible with ultrasound, may be used as the suprascapular notch landmark for procedures around the suprascapular notch region.

## Figures and Tables

**Table 1:** Distribution of the suprascapular notch types in the whole group including body sides.

Type of suprascapular notch	All (*n* = 235)	Right (*n* = 116)	Left (*n* = 119)
I [*n* (%)]	26 (11.1%)	13 (11.2%)	13 (10.9%)
II [*n* (%)]	14 (6.0%)	6 (5.2%)	8 (6.7%)
III [*n* (%)]	151 (64.0%)	76 (65.5%)	75 (63.0%)
IV/V [*n* (%)]	44 (18.7%)	21 (18.1%)	23 (19.3%)
Level *p*	-	0.9512	

**Table 2:** Distribution of the visualization of the suprascapular vein depending on suprascapular notch type.

Type of suprascapular notch	I [*n* (%)]	II [*n* (%)]	III [*n* (%)]	IV/V [*n* (%)]
Suprascapular vein visible (+)	2 (7.7%)	4 (28.6%)	130 (86.1%)	40 (90.9%)
Suprascapular vein not visible (−)	24 (92.3%)	10 (71.4%)	21 (13.9%)	4 (9.1%)
*p* value	**0.0001**			

**Table 3:** Distribution of the visualization of the suprascapular nerve depending on suprascapular notch type.

Type of suprascapular notch	I [*n* (%)]	II [*n* (%)]	III [*n* (%)]	IV/V [*n* (%)]
Suprascapular nerve visible (+)	2 (7.7%)	6 (57.14%)	67 (44.37%)	38 (86.36%)
Suprascapular nerve not visible (−)	24 (92.3%)	8 (42.9%)	84 (55.6%)	6 (13.6%)
*p* value	**0.0001**			

**Table 4:** Dimension of suprascapular notch and visualization of the suprascapular nerve.

Dimension of suprascapular notch	Suprascapular nerve visible (+)	Suprascapular nerve not visible (−)	*p* value
Superior transverse diameter [mm]	13.2 +/− 4.1	15.6 +/− 4.4	**0.0027**
Maximal depth [mm]	5.8 +/− 1.7	7.6 +/− 2.6	**0.0001**
